# Facilitators and Barriers of Adolescent Self‐Disclosure Across Different Confidants: A Multi‐Informant Mixed Methods Study

**DOI:** 10.1002/jad.70100

**Published:** 2026-01-05

**Authors:** Marie‐Louise J. Kullberg, Loes Keijsers, Bernet Elzinga, Loes H. C. Janssen

**Affiliations:** ^1^ Department of Clinical Psychology Leiden University Leiden the Netherlands; ^2^ Department of Psychology, Education, and Child Studies Erasmus University Rotterdam Rotterdam the Netherlands; ^3^ Leiden Institute for Brain and Cognition (LIBC) Leiden University Leiden the Netherlands; ^4^ Department of Developmental Psychology Utrecht University Utrecht the Netherlands

**Keywords:** adolescents, mixed methods, parents, peers, self‐disclosure, trusted adults

## Abstract

**Introduction:**

Adolescent self‐disclosure is essential for relationship building, receiving support and mental well‐being. However, little is known about whom adolescents confide in and what factors facilitate or hinder this process.

**Method:**

In this mixed methods multi‐informant study, twelve Dutch adolescents (*M*_age_ = 18.3, 66.6% girls) and their parents (11 fathers, 12 mothers) were interviewed. Inspired by Q‐methodology, adolescents placed color‐coded cards on a grid to indicate whether they discussed 16 potentially distressing topics (e.g., mental health, school problems, physical complaints, family issues) with 17 persons (five relationship categories: caregivers, peers, professionals, familiar adults and others).

**Results:**

Quantitative results show that adolescents disclosed most to caregivers (especially mothers) and least to familiar adults, such as teachers. Semistructured interviews with adolescents and their parents were analyzed using inductive reflexive thematic analysis. Warm relationships and concrete opportunities to talk facilitated self‐disclosure. Barriers included emotional distance and privacy concerns. Parents reported dilemmas between initiating conversations with their child and allowing space.

**Conclusion:**

Adolescents appear to be willing to share distressing topics, but familiar adults may need to take a more active role in fostering disclosure by making time, creating an inviting atmosphere, and initiating conversations.

## 1 Introduction

Adolescents navigate a period of biological, emotional, and social changes that can be both exciting and challenging. Experiencing physical changes, mood swings, and evolving relationships—although entirely normal—can make adolescents vulnerable to emotional distress (Dahl et al. 2018; Rapee et al. 2019). One important strategy for managing everyday difficulties is to seek social support, which is only possible if adolescents share personal or sensitive thoughts, venting, feelings, experiences, and beliefs with others with others, a process known as *self‐disclosure* (Petronio 2002; Tilton‐Weaver et al. 2014; Vijayakumar and Pfeifer 2020). Self‐disclosure involves voluntarily sharing meaningful, personal information and is distinct from routine disclosure, such as sharing information about activities and whereabouts (Tilton‐Weaver et al. 2014). Self‐disclosure is key to healthy development in adolescence: It enhances psychological well‐being, acts as social glue, facilitates interpersonal communication and the formation of reciprocal relationships, supports identity formation, and advances self‐worth (Smyth et al. 2012; Vijayakumar and Pfeifer 2020). Therefore, this study examines how adolescents manage self‐disclosure in various types of relationships, including parents, friends, but also other familiar adults, such as teachers, professionals. To whom do adolescents disclosure their concerns and worries? And what barriers do they experience?

### 1.1 Adolescents' Self‐Disclosure to Familiar Persons

Adolescents tend to confide less in those around them as compared to adults, who often share most of their daily experiences to close others (Rimé et al. 1998). Adolescents are still developing their ability to decide whether or not to disclose, and who to confide in, by weighing the risks and benefits of sharing personal information (Tilton‐Weaver and Marshall 2008; Elsharnouby and Dost‐Gözkan 2020). Even though approximately 80%–95% of adolescents have someone in their social network they can turn to for support (Mirković and Merkaš 2019; Rhodes and DuBois 2008), adolescents also vary whom they would approach. For instance, a recent study among 188.000 Dutch adolescents found that 81% would turn to a parent when they face difficulties, 65% would turn to friends, 35% to a family member, 20% to a trusted adult at school, and 8% would turn to a professional (GGD Nederland 2024).

This extension of adolescents' social network is an important developmental task. With regard to their parents, it is healthy and normative for adolescents to establish independence. One way of doing so is to set personal boundaries around sensitive topics, such as emotions and bodily experiences (Petronio 2002). This individuation process typically results in reduced disclosure to parents and increased secrecy (Finkenauer et al. 2002; Keijsers and Poulin 2013). While friends increasingly become essential confidants during adolescence (Frijns et al. 2013), there are also significant barriers to sharing with friends, such as lack of trust or shame. Relationships in the broader social network, such as siblings, older peers, or nonparental, neutral outside the family (“familiar adults”) may become more crucial, and are sometimes even preferred over more close friends for discussing certain sensitive topics due to perceived confidentiality and neutrality (Chen and Danish 2010; Villalobos Solís et al. 2015).

Prior work has suggested that self‐disclosure to one's broader social network is an important strategy in managing difficulties and contributes to adolescents' mental well‐being (Smyth et al. 2012; Vijayakumar and Pfeifer 2020). However, a significant limitation of previous research is that adolescent self‐disclosure has primarily been examined in relation to parents and has been understudied in the context of other close relationships (e.g., siblings, peers, familiar adults; Guo et al. 2022; Finkenauer et al. 2024). A more comprehensive understanding of (1) to whom adolescents disclose (e.g., caregivers, peers, relatives and familiar adults and others) and (2) the facilitators and barriers to adolescent self‐disclosure is lacking and represents the key objective of this study. To address this objective, we employed a convergent parallel mixed methods design. The quantitative approach for Aim 1 was used to measure how frequently adolescents disclose information and to whom, providing an overview of disclosure patterns. The qualitative approach for Aim 2 aimed to explore the reasons behind these behaviors, offering rich, contextual insights into facilitators and barriers of self‐disclosure. This mixed methods design was chosen to combine the strengths of both approaches. Data were collected in parallel sessions, but analysis and discussion were conducted sequentially.

### 1.2 Adolescents' Perception on Responses to Their Self‐Disclosure

One of the specific barriers and facilitators that we examine is the anticipated response of the other, particularly how adolescents perceive the responses, as these may shape the self‐disclosure process. Adolescents often decide to disclose in order to organize their thoughts, vent, or receive support (Duprez et al. 2015). The response they receive plays a crucial role in whether or not these needs are met, thereby shaping the likelihood of future disclosures. Studies focusing on adolescents' perspectives on parental responses to self‐disclosure have shown that adolescents view reactions such as expressing sadness or worry as undesirable (Darling et al. 2006; Tokić and Pećnik 2011) and emotional and instrumental support from parents (Tokić and Pećnik 2011). In line with this, an observational study found that high levels of parental validation were associated with earlier emotional disclosure by adolescents during a 10‐min conflict discussion (Main et al. 2019). However, existing studies on responses to adolescent self‐disclosure predominantly focused on parental responses. This study aims to extend the existing knowledge on adolescents' perceptions of responses by also exploring adolescents' perceptions of responses from friends, other family members, and familiar adults.

### 1.3 Parental Self‐Disclosure as Modeling Process

During adolescence, adolescents and parents undergo a transformation in their relationship, with parents remaining a crucial source of support (Branje 2018). In this light, we focus specifically on how self‐disclosure may function in this caregiving context (Smetana et al. 2006). Adolescent's willingness to disclose may be shaped by parents own self‐disclosure, but parental self‐disclosure and the impact on adolescent self‐disclosure remains largely unexplored. The process of modeling is well‐established in the coping literature. Parents play a crucial role in modeling adolescents how to cope with emotions and distress (Abaied and Rudolph 2010; Kliewer et al. 1996; Miller et al. 2010). Children implicitly learn coping behaviors by observing their parents and subsequently apply these strategies in their own lives, often without direct guidance from the parent (Kliewer et al. 1996; Zimmer‐Gembeck and Locke 2007). How mothers and fathers themselves cope with emotions and stressful events is directly related to the child's coping strategies (Liga et al. 2020). Therefore, we also explore parents' perspectives on their own self‐disclosure to potential confidants and how they think it influences their adolescents' self‐disclosure.

### 1.4 The Present Study

The aim of the current study is to explore the facilitators and barriers for adolescent self‐disclosure to various relationships (caregivers, relatives and familiar adults, peers, professionals, and others), given that self‐disclosure is key to healthy development (Smyth et al. 2012). Using a mixed methods design, we answered four research questions: RQ1) To whom do adolescents disclose different types of distressing topics? RQ2) What are facilitators and barriers of adolescents' self‐disclosure with specific persons in their social environment? RQ3) How do adolescents perceive the responses from others to their self‐disclosure? RQ4) What are parents' perspectives on their own self‐disclosure and the impact on adolescent self‐disclosure? Given the exploratory nature of the study, no hypotheses were specified. To answer these questions, we included adolescents from the larger, Dutch multimethod two‐generation RE‐PAIR (“Relations and Emotions in Parent‐Adolescent Interaction Research”) study which included adolescents with and without depression (Janssen, Verkuil, van Houtum et al. 2024).

Answering the questions as formulated above could provide insights into how adolescents' social context can better facilitate self‐disclosure. Ultimately, it may help to alleviate the strain on mental health care systems, as adolescents pointed out that having access to trusted figures, such as teachers, coaches, or older peers, could reduce their need for professional support (Common Good Labs 2024).

## 2 Methods

### 2.1 Design

**Figure 1:**
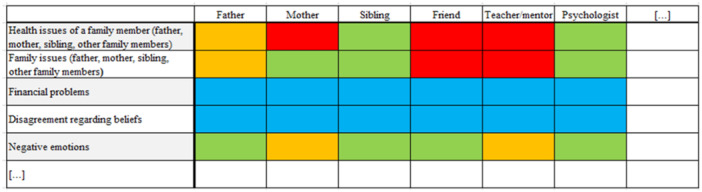
An example of parts of a completed matrix.

**Figure 2:**
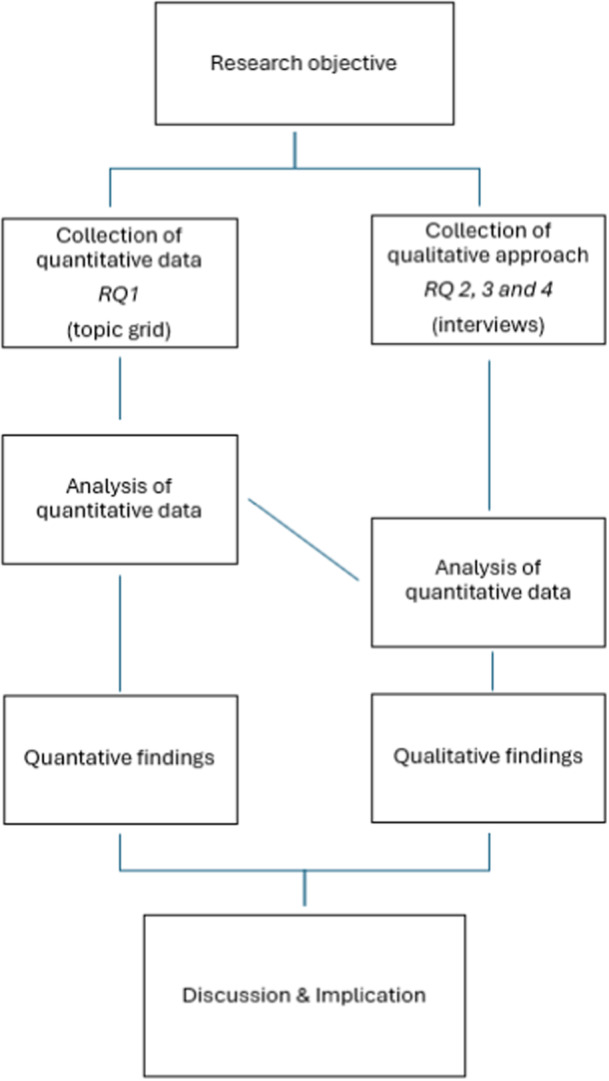
Design chart.

A convergent parallel mixed methods design was employed to provide both breadth and depth of insights into adolescent self‐disclosure and to capture the lived experiences of adolescents and their parents that cannot be captured through standardized instruments. Data was collected in parallel, but analysis was conducted sequentially. To obtain a comprehensive picture of all potentially relevant confidants to whom adolescents disclose and of what they share with familiar persons, we developed and used a topic grid to assess disclosure to potential relevant confidants about topics, covering 17 potentially trusted persons and 16 potentially distressing topics, see Figure 1 for an example, the full blank grid can be found in Appendix S1. By displaying a wide variety of topics and potential interaction partners on the topic grid, it allowed us to discuss topics and people adolescents would not have thought of themselves. Facilitators and barriers of adolescent self‐disclosure (2), perceived responses, (3) and parents' perception on own self‐disclosure (4) were addressed qualitatively in a semistructured interview with adolescents and both mothers and fathers. Figure 2 depicts the chart of our convergent parallel mixed‐methods design, which illustrates how the quantitative and qualitative components are paralleled and integrated.

### 2.2 Participants

The study used purposive sampling, which means that participants were selected because they were able to articulate a viewpoint on the topic (Stenner et al. 2008; Tongco 2007). Adolescents—without psychopathology and with a depressive disorder as determined with a clinical interview—and their parents, who participated in the Dutch multi‐method two‐generation RE‐PAIR (“Relations and Emotions in Parent‐Adolescent Interaction Research”) study and who gave consent to be contacted for follow‐up studies were invited to participate in the current study (see for more information Janssen, Verkuil, Nedderhoff et al. 2024). Inclusion criteria for the adolescents to participate in the current study were: age between 12 and 21 years and having a good command of Dutch. A total of 12 Dutch adolescents and 23 of their parents or primary caregivers (all White) participated. The adolescents ranged in age from 15 to 21 years, with a mean age of 18.3 (SD = 1.70). Four out of the 12 (33.3%) were male. Ten adolescents lived with one or both of their parents and two adolescents lived on their own. Of the 23 parents, 11 parents were male (52.1%). Three of the adolescents entered RE‐PAIR as adolescents with a depressive disorder, the others nine entered as adolescents without psychopathology. The mean Patient Health Questionnaire (PHQ‐9; Kroenke et al. 2001) sum score of all adolescents was 9.17 (SD = 7.90, range: 0–25), of which 3 adolescents scored above the threshold of 10, suggesting the presence of a current depression (Manea et al. 2012). Descriptive statistics of the adolescents are presented in Table 1.

**Table 1:** Descriptive characteristics of the adolescents.

Adolescent pseudonym	Adolescent age (years)	Adolescent gender	‍Adolescent depressive symptoms (PHQ‐9)	Participated with mother	Participated with father
Ravi	17	Male	‍7	Yes	Yes
Sebastian	16	Male	‍4	Yes, but no transcript	Yes, but no transcript
Leah	19	Female	‍22	Yes	Yes
Sofia	19	Female	‍16	Yes	Yes
Layla	19	Female	‍9	Yes	Yes
Jim	21	Male	‍0	Yes	Yes
Chloe	17	Female	‍25	Yes	Yes
Amy	19	Female	‍8	Yes	Yes
Omar	20	Male	‍3	No	Yes
Olivia	18	Female	‍5	Yes	Yes
Nia	15	Female	‍9	Yes	Yes
Indira	20	Female	‍2	Yes	Yes

### 2.3 Procedure

Data collection took place between December 2022 and March 2023; either face‐to‐face in families' homes or online via Microsoft Teams. Families could choose whether they preferred a home visit or an online session (*n* = 3 families face‐to‐face; *n* = 9 families online). Two authors (M. K. and L. J.) conducted all interviews and each interview started with a general introduction of 10 min for the adolescent and parents. They then split up, with one researcher interviewing the adolescent and the other researcher interviewing the parent(s). If both parents were available, they were interviewed one by one. In the interviews with the adolescents, they were first asked how they would describe moments when they are not feeling well and how they notice it, in order to use the adolescents' own wording during the interview to match their lived experiences. Next, the researcher gave additional instructions about the grid and semi‐structured interviews.

All sessions were recorded using Microsoft Teams with the transcription mode enabled. All transcripts were cleaned and checked for accuracy by Psychology Master students. Recording of one parent couple was corrupted and could not be transcribed, resulting in *n* = 21 parent interviews. Interviews with adolescents, which included completing the grid and answering qualitative questions, took 30–90 min. Parent interviews lasted 20–70 min.

After participation, parents and adolescents were debriefed and received 15 and 10 euros respectively. The study was approved by the Research Ethics Committee at Leiden University (2021‐10‐12‐B.M.Elzinga‐V2‐3476). The data underlying this article cannot be shared publicly due to ethical permissions. Before starting the individual interviews, participants first provided informed consent and adolescents completed a short questionnaire on their depressive symptoms (PHQ‐9). If adolescents were below 16, parents also provided informed consent for their child. The data underlying this article canno.

### 2.4 Measures

#### 2.4.1 Topic Grid and Semistructured Interviews

To obtain an in‐depth understanding of to whom adolescents disclose about distressing topics (RQ1), we used a topic grid inspired by the Q‐methodology (see Figure 1). The grid represented common distressing topics and potential confidants in adolescents' lives. In designing the grid, we used an iterative approach to ensure that the grid contained an overview of relevant persons and topics. The research team initially designed a grid that included topics and persons based on scientific literature on adolescent development (Dahl et al. 2018; Rapee et al. 2019), social domain theory (Smetana et al. 2009), and the researchers' clinical experience. The grid was reviewed by four master's students closely in age to the sample, who suggested adjustments based on their own lived experiences as adolescents. Adjustments were mainly textual and some examples were added. The final complete grid consisted of 256 cells, representing of 16 common and important topics for adolescents (e.g., mental health problems, financial concerns, including an “other” option; see Table 2 for an overview) and 17 potentially trusted persons (e.g., father, mother, peer, including an “other” option). An example of parts of a completed matrix is shown in Figure 1, the full blank grid can be found in Appendix S1.

**Table 2:** Overview of the discussed topics ordered as in the grid.

Topics	Examples of discussed topics
‍Health issues of a family member (father, mother, sibling, other family members)	Family member with cancer; mental health issues of family member
Family issues (father, mother, sibling, other family members)	Divorce parents; tension between parent and sibling
Financial problems	Earning money; financial concerns
Disagreements regarding beliefs	Different political opinion; religion; social, political, faith
‍Negative emotions	Irritation; not feeling well; sadness, stress
‍Mental health problems	Panic attics; anxiety; depressive symptoms; suicidality, self‐harm
Issues related to school	Stress; lack of motivation; grades; exams
‍Bullying, exclusion, racism, intimidation	Exclusion at school, bullying
Issues with friends/friendships	Fight with a friend; breaking off friendships; discussions, friend in trouble, peer pressure
Issues with partner/romantic relationships	Breaking up; never had a relationship; insecurity, irritation, sexuality
Bodily issues	Weight insecurity; piercings; change in appearance
Identity issues	Sexual orientation; gender
Social media issues	Amount of use (screen time); addiction; disturbing messages, unwanted content, amount of use
Health issues	Bad sleeping; surgery; eating, sleeping, illnesses
Issues with alcohol, drugs, smoking, gaming	Own use; use of others (friends)
Other negative events	Suicide of acquaintance, grandparent that died unexpectedly

Participants were guided through the grid by the researchers. The researcher asked the participants, topic by topic, if they had experienced something like this and if they would talk about it with each person on the grid. For each of the 16 topics, adolescents were asked to indicate with whom they would discuss the topic by placing a colored card on the grid. Adolescents had three answer‐options. A green card represented “I would definitely discuss this topic with this person,” the red card represented “I would definitely not discuss this topic with this person,” and orange represented “I would perhaps discuss this topic (if this person would ask about it).” If adolescents did not have any issues with regard to a certain topic (e.g., there are no monetary issues) or did not have a stepmother, a blue card was selected representing “not applicable.” When the data collection took place online, researchers shared their screen, displayed the grid, and colored the boxes in response to adolescents' answers.

Subsequently, per card, the adolescent was asked the following questions: (RQ2) why they would or would not talk about this topic with this given person to assess (2a) barriers and (2b) facilitators per topic and per person. Related to RQ3, if a green card was placed, we also asked (3) how adolescents perceived the response of the other person.

To answer RQ4 semistructured interview were held with parents of the adolescents. As introductory questions, parents were asked how they notice that their adolescent is not feeling well, how they deal with moments when their adolescent is not feeling well, and to whom they think their adolescent discloses. The main question was what the parents themselves did when they did not feel well.

#### 2.4.2 Depressive Symptoms

The Patient Health Questionnaire (PHQ‐9; Kroenke et al. 2001) was used to screen online for the presence of depressive symptoms in the past 2 weeks prior to the data collection. The items are based on nine DSM‐IV criteria for depression and are scored from 0 (*not at all*) to 3 (*nearly every day*). One item (“moving or speaking slowly or being so fidgety or restless”) was split into two items and the maximum score of these two items was included in line with RE‐PAIR. Total scores ranged from 0 to 27, with scores above 10 suggesting the presence of depression (Manea et al. 2012). Cronbach's *α* was 0.96 indicating a good internal consistency of the PHQ‐items.

### 2.5 Data Analysis

#### 2.5.1 Quantitative Analysis

To answer RQ1 “to whom do adolescents disclose about distressing topics?” and to gain insight into who adolescents do and do not disclose to, we calculated the frequencies of each response category (always [green], sometimes/maybe if asked [orange], never [red], not applicable [blue]) for each topic and each person across all participants. Response frequencies were calculated per interaction partner in Excel. For example, we calculated the frequency of *always* self‐disclosing to their mother about emotions, *never* self‐disclosing to their mother about emotions, etc. We controlled for the “not applicable” response option. Next, we calculated the average frequency of disclosing about the 16 topics to a specific person by dividing the summed percentages of always talking to person X about to topic Y by the number of topics (16). We interpreted these frequency scores and did not compare between confidants using significance tests.

#### 2.5.2 Qualitative Analysis

After the quantitative analysis, we used inductive reflexive thematic analysis of the interviews with adolescents and their parents to answer RQ2 to 4 (see also Figure 2; Boeije and Bleijenbergh 2019). This is a well‐established qualitative, interpretatively focused approach that is commonly used to analyze qualitative data (Braun and Clarke 2012; Thompson et al. 2022). The analysis involved several steps. First, M. K. and L. J. read the transcripts of interviews with adolescents and identified sections of the interview related to three domains. To answer RQ2, the (a) facilitators, why adolescents would discuss a topic with a person and (b) barriers, why adolescents would do not discuss a topic with a person, were selected. To assess RQ3, adolescents' perceptions of responses of persons were selected. Finally, to answer RQ4, about parents' perception on own self‐disclosure, MK and LJ read the transcripts of the interviews with parents and identified sections on parents' perspectives on their own self‐disclosure and the impact on adolescent' self‐disclosure. Second, M. K. and L. J. continued to open code all of the interviews together to reach full consensus. This exploration phase resulted in the definition of initial codes. Third, M. K. and L. J. generated initial themes based on the coding using axial coding. Lastly, with the identified themes in mind, M. K. and L. J. reviewed the given codes and refined, defined, and (re)named the themes. As reflexivity is a critical element of reflexive thematic analysis (Braun and Clarke 2019), we reflect on the roles of the researchers. The study team consisted of researchers with expertise in adolescents, mental health, well‐being, and parenting. M. K. also works as a psychologist in an academic mental health setting. In presenting and discussing the findings from our qualitative analysis, we focus on the confidants most commonly identified by adolescents in our quantitative analysis. These include parents, siblings, friends, partners, teachers, health professionals (merging psychologist, general practitioner, other professionals), and internet (merging others online and searching information).

## 3 Results

### 3.1 Quantitative Analysis

#### 3.1.1 Who Do Adolescents Disclose To?

To answer RQ1, to whom do adolescents disclose about distressing topics, the frequencies of the Q‐grid data were calculated. An overview of how often adolescents disclose to persons in their direct environment, averaged across all topics, is presented in Table 3. Overall, for the “always” response category, we found that most of the topics were disclosed to mothers (75.6%), followed by others including partner, colleagues, and classmates (54.8%), friends (51.8%), psychologists (48.5%), and fathers (44.7%). Many topics were also discussed with stepfathers if present (59.4%), but this only concerned two adolescents. Adolescents were least likely to discuss the topics (response option “always”) with neighbors/family acquaintances (0%), stepmothers and stepsiblings (0%), teachers (10%), and general practitioners (10.3%). To further clarify, we grouped the persons into five categories (caregivers, peers, relatives and familiar adults, professionals, and others). A full overview of how often adolescents always, never, and sometimes disclose by topic is presented in Appendix S2–S4.

**Table 3:** Frequencies (%) of adolescent self‐disclosure to others averaged over topics.

	Number of adolescents^a^	Always (%)	Sometimes/maybe (if asked) (%)	Never (%)	Examples of discussed topics
*Caregivers*					
Mother	12	75.6	15.4	16.3	Family issues, financial problems, disagreements about beliefs
Stepfather	2	59.4	9.4	12.5	Health issues, Family issues, disagreements about beliefs
Father	11	44.7	29.8	25.5	Disagreement about beliefs, issues related to school, other negative events
Stepmother	2	0.0	31.3	56.3	Family issues, mental health problems, issues with friends
*Peers*					
Friend	12	51.8	25.1	23.1	Issues with partner/romantic relationship, social media issues, issues with alcohol, drugs, smoking, gaming
Sibling	11	34.1	30.1	35.8	Disagreement about beliefs, negative emotions, other negative events
Step sibling	2	0.0	3.1	78.1	Health issues family
*Relatives and familiar adults*					
Grandmother/grandfather	11	7.3	18.4	74.3	Other negative events, health issues family, disagreement about beliefs
Uncle/aunt	12	4.2	18.6	77.2	Social media issues, other negative events, health issues family
Neighbors/family acquaintances	12	0.0	0.0	81.3	
*Professionals*					
Psychologist	9	48.5	24.8	26.6	Mental health problems, negative emotions, issues related to school
Other professional	3	34.9	16.7	42.2	Financial problems, health issues family, family issues
General practitioner	12	10.3	7.5	82.3	Mental health problems, health issues, other negative events
Teacher/mentor	12	10.0	19.4	70.7	Issues related to school, health issues family, negative emotions
*Others*					
Other, namely	11	54.8	13.9	31.3	Issues with alcohol, drugs, smoking, gaming, issues related to school, negative emotions
Search information online	12	23.1	25.1	51.8	Health issues, mental health problems, identity issues
Others online	7	0.7	4.4	94.9	Issues with friends, disagreements regarding belief, health issues family

*Note:* The “other, namely” category included, partner, colleague, class mate, the person I am dating, God. Frequencies were controlled for the “not applicable” response option and therefore do not add up to 100%.

^a^ Refers to number of adolescents who talked about these persons (excluding “non‐applicable” option).

#### 3.1.2 About What Topics Do Adolescent Disclose?

Topics were explored in an exploratory manner to gain some insight into topics that were discussed always with certain persons.

##### 3.1.2.1 Caregivers

All adolescents shared at least one topic with their mothers. Adolescents disclosed about a wide range of topics, including family‐related issues (both health and general), financial problems, negative emotions, issues related to school, and issues with partner/romantic relationship. Issues with alcohol, drugs, and smoking were discussed the least with mothers. Adolescents also discussed a wide range of issues with fathers, including beliefs, financial problems, school, health in the family, and relationships with friends. Adolescents were less likely to talk to fathers about issues related to mental health, with partner/romantic relationship, identity, and social media. Interestingly, the adolescents who had a stepfather (*n* = 2) disclosed to him about almost all topics, expect for bodily issues.

##### 3.1.2.2 Peers

Adolescents disclosed to friends about a wide range of issues, including health in the family, mental health problems, school, identity, friends, partner/romantic relationship, social media, and alcohol, drugs, smoking, and gaming. Adolescents were less likely to disclose to friends about family‐related issues, financial problems, and bodily concerns. To their siblings, adolescents talked about beliefs, negative emotions, health issues in the family, family issues, and other negative events. Bodily issues, identity issues, issues with partner/romantic relationship, bullying, and financial problems were not often discussed.

##### 3.1.2.3 Relatives and Familiar Adults

When adolescents disclosed their worries and concerns to an aunt/uncle, it was about social media, other negative events, and health in the family. For grandmother/grandfather, adolescents disclosed about other negative events, health issues in the family, and disagreements about beliefs. Adolescents did not disclose to neighbors/family acquaintances.

##### 3.1.2.4 Professional

At the screening, nine out of 12 adolescents reported that they had recently seen a psychologist (75%). With regard to the psychologist, adolescents disclosed about mental health problems, negative emotions, issues related to school, health issues in the family, family issues, bullying, issues with partner/romantic relationship, own health issues, and (other) negative events. Issues related to relationships with friends, identity, and social media were not discussed with the psychologist. When adolescents disclosed to the general practitioner, it was about mental health problems and health problems. With teachers or mentors, adolescents shared issues related to school and sometimes health issues in the family.

##### 3.1.2.5 Others

Adolescents disclosed to the “other” category, including a partner, colleague, class mate, the person I am dating, and God on a wide range of topics. The topics include issues with alcohol, drugs, smoking, gaming, school, family (health and general), negative emotions, partner/romantic relationship, health, and friends. Bodily issues were not shared with others. Adolescents searched for information about health issues, identity issues, and mental health problems, but did not discuss any of these topics with others online.

### 3.2 Qualitative Analysis

We analyzed the interviews with the adolescents on the predefined domains related to our research questions RQ2 and RQ3: (2a) Facilitators of sharing; (2b) Barriers of sharing; (3) Adolescents' perception of responses of others. For each domain, we identified themes through thematic analyses. Based on the quantitative analyses, we focus on the most common people adolescents disclose to, namely parents, siblings, friends, partners, teachers, health professionals (merging psychologist, general practitioner, other professionals), and internet (merging others online and searching information) in the discussion of our qualitative analyses. Finally, as the main source of support for many adolescents, we analyzed the interviews of the parents to answer RQ4 “what the perception of adolescents' parents is on their own self‐disclosure and the impact on their adolescents' self‐disclosure.”

#### 3.2.1 Facilitators of Sharing

##### 3.2.1.1 Initiation of the Conversation by a Confidant

Adolescents reported that they were more inclined to share or discuss distressing topics when mothers, fathers, siblings, or partners initiated the conversation and provided an opportunity, which can also be referred to as a “partner effect.” For instance, Sebastian indicated that he discussed emotions with friends when they asked about it: “Yes, they ask about it because they see you, they ask why I look a certain way, or something, but I wouldn't start about it myself, but if they ask about it, yes.” This facilitator was also noted by adolescents for teachers. Jim reported that his mentor asked about the bullying he had experienced: “Because they asked about it, they gave an opening.”

##### 3.2.1.2 Warm Relationship

A warm and intimate relationship emerged as a key facilitator for discussing distressing issues with mothers, fathers, siblings, partners, and friends. For instance, because of the close connection Layla feels with her sibling, she discussed the heavy alcohol use of some friends with him: “We have quite an open relationship I think. We can share and say a lot to each other.” Amy talks about her emotions with her friends because she knows they will listen to her and are very involved: “With one, say, two friends from high school I discuss literally everything in terms of emotions and things like that. I like those two friends much better because I can just tell my story to them and they really listen and ask questions and stay involved.” With friends, physical proximity also facilitated conversations about distressing topics.

##### 3.2.1.3 Shared Experiences

Shared experiences with parents, siblings, and friends, such as stress of exams, illness, or the death of a relative, also facilitated talking about distressing topics, because the adolescents felt that the other person could relate. Sofia's grandfather died, which she discussed with her parents: “…when you're in the middle of it, in the process of going to the funeral, then you really do talk about it a lot with each other. Also about how everyone feels about it.” Similarly, Olivia's grandparents had dementia and passed away, which she talked about with her friend: “my friend's grandfather who had dementia too, also died recently so we were kind of in the same trajectory.”

##### 3.2.1.4 Adolescent's Emotional or Practical Need

Adolescents' own motivation also facilitated their self‐disclosure. When driven by an emotional need (e.g. to feel supported, wanting to vent/share etc.), adolescents turned to their fathers, mothers, siblings, friends, or partners. Layla also turned to God: “it gives me a lot of peace of mind to just have some kind of constant connection.” Adolescents also share worries with, for instance parents or a sibling, to inform them. Layla tells her parents about the smoking and alcohol use in her group of friends “These are things that I mention to my parents, but mainly that they know what I am up to.”

With regard to a practical need (e.g., for advice or a solution), adolescents turned to teachers or professionals mainly for functional reasons rather than for emotional need. For example, to get a referral letter from the general practitioner for psychological help, or to discuss at school why they had missed a deadline or class. For instance, Olivia her grandparents died unexpectedly: “They should know why I do not attend class.”

##### 3.2.1.5 Emotional Urgency

Adolescents' motivation to disclose increased with emotional urgency. They reported only discussing topics that really bothered them or were highly emotional, especially with fathers and friends. Nia talked to her friends about her father cheating on her mother: “… I had a conversation with one of my friends and I blurt it out. But yeah, you know, it's also something you don't want half the world to know, it gives you a reputation.” Similarly Olivia discussed her insecurities about her body with some friends “… I did talk about it the other day, about how I really felt very insecure at the time.” Amy talked with her father about her history of being bullied in primary school: “At some point it [the story] all came out, but not when it was happening all along.”

##### 3.2.1.6 Other Aspects Related to Adolescent Self‐Disclosure

Finally, adolescents shared some of their own capabilities and strategies for self‐disclosure. Adolescents occasionally broached topics with fathers and friends if they were able to do so superficially or with humor, such as Leah: “I used to be pretty much just keeping everything to myself, but I did just learn to just discuss then with certain friends and also just friends that I trust. So I do discuss it with them, but also just like that, just as a joke or something. Because I do use humor often with them. So I do go there often when I don't feel good.” Adolescents only used the internet to seek information on specific distressing topics (e.g., on how to lose weight, about a planned surgery, how to distract from a negative mood). Indira for instance mentioned: “I was just more looking for, like, (…) how do I lose weight and, I don't know, dieting and whatever.” Adolescents also looked online for shared experiences (e.g., having suicidal thoughts, mental health problems) such as Layla who spoke about dealing with emotions: “(…) You happen to come across those groups in which other people are also talking about things they've experienced and how they feel about them.”

#### 3.2.2 Barriers in Sharing

##### 3.2.2.1 Personal Matter and Privacy

An important barrier for adolescents in self‐disclosing distressing topics to adults, including fathers and mothers, but also teachers, was the personal nature of these matters. Olivia felt lonely during the COVID‐19 pandemic and never discussed it with her parents: “I guess I just didn't want to, I just wanted to keep it to myself. Maybe I wanted to in the moment but I never did.” Amy did not want to talk to her mentor at school about her mom's health problems: “because those people are pretty far away for me, so then when something personal like that happens then, yeah, I don't know, there's just kind of a blockage.” Privacy was also a significant barrier with friends. Amy did not want to talk to her friends about the conflict her parents were having: “That's just something within the family I think, and I don't think you should discuss that so much with people outside the family.” In the context of privacy and viewing the issues as a personal matter, emotional distance and a lack of opportunities to discuss such issues were cited as specific barriers for teachers. For example, Amy did not discuss her mental health problems with her teacher: “Because for me that's just something very personal, and a teacher of mentor feels too distant to me.”

##### 3.2.2.2 Protecting the Other

Many adolescents avoided self‐disclosing about distressing topics to their parents to protect them from the emotional burden, such as Leah: “After my previous suicide attempts, I am also just afraid of hurting them even more and making them anxious, because they are also already quite worried.” Similarly, Amy refrained from talking to her father about her mother's health problems: “Because I know he's concerned about it too, so then I think I'll keep that to myself.”

##### 3.2.2.3 Shame and Taboo

Shame was mentioned as a complicating factor in discussing distressing topics with friends and also with adults, such as family and teachers/mentor. Indira gained weight during the period when she was not doing well mentally and did not discuss this with her friends. “It is quite personal, you feel a bit ashamed of it too, so it did not feel good [to discuss].” Leah was worried about her financial situation and felt ashamed to discuss it with her friends “Because then I'm kind of afraid, yeah I don't know, I think maybe it's a little embarrassing or something, because nobody really talks about it. […] A couple of my friends once had a 50 euro beer bill for one evening and then I really think, yes, you can't say you're poor, because that, you know, that's just two weeks of groceries.” In addition to shame, existing norms or a topic being taboo prevented adolescents from disclosing, for instance feelings of stress and worries about school, such as Ravi: “Yeah you don't talk [with friends] about your internal feelings or stress or anything. More like what happens in the external world.”

##### 3.2.2.4 Expected Negative Response

The anticipation of a negative response inhibited adolescents from talking about distressing topics with fathers, mothers, siblings, friends, professionals, and using the internet. For instance, Amy mentioned: “But I also have a friend who I have actually known all my life, with whom I discuss it comparatively a lot less. I mention it sometimes, but I don't really discuss it because the conversations goes quickly about her [friend] emotions, that I find difficult.” Specifically for partners and professionals, some adolescents cited past negative experiences (including violations of privacy and confidentiality) that discouraged them from future discussions of emotional topics, such as Nia: “I don't like her [the school psychologist] very much, not because she is not nice. Just because I have noticed with several of my friends that she passes things on to parents.”

##### 3.2.2.5 Lack of Opportunity

The lack of opportunity, such as the lack of physical presence, limited time together, or not feeling space to discuss issues, was also mentioned as a barrier, for instance with fathers, friends and teachers, but interestingly not for mothers. Sofia mentions her father: “But he is still more involved in sports or something in his free time. He is also working more hours and leaving early for work.”

##### 3.2.2.6 Deemed Unimportance

Additionally, adolescents often did not feel the need to share distressing issues with mothers, friends, and professionals because they considered the issues unimportant or not serious enough. Ravi, for example, mentioned his mental health issues, which he did not share with his parents: “At first, I didn't even realize that was a thing, you know, I just thought it was normal.”

##### 3.2.2.7 Alternative Coping Strategies or Confidants

Adolescents refrained from discussing distressing topics with their parents because they had alternative coping strategies, such as talking to someone else or dealing with the situation independently. Amy did not want to talk about her mental health issues with her parents “…I already share it with other people, I talk about it then. I didn't really feel that my parents would be able to help well with it […].”

#### 3.2.3 Adolescents' Perception on the Responses of Others

Given that self‐disclosure is inherently a social phenomenon, a third research question was how adolescents' perceived others' responses to their self‐disclosure. When adolescents discussed distressing topics, they most often received supportive responses from all persons in their social network, including caregivers, peers, relatives and familiar adults, professionals, and others. Amy for instance reported on her sister: “Very supportive. She gives me a big hug when I am very stressed or sad. She takes a break and lets me tell my story and asks supportive questions. With her, it's just that I can really tell my story.” However, adolescents also noted that discussing distressing topics sometimes led mothers, siblings, and partners to become emotional themselves (i.e., emotional transmission)—a response they sometimes perceived as caring, but also sometimes found challenging. Amy indicated that when she shared that she was upset about tension between her mom and dad that her mom became emotional “On the one hand, I get it, because she blames herself […]. It is not how I want her to react, it makes me feel guilty for saying it to her.”

Both fathers and teachers were noted for their practical responses and advice, as Sofia indicated: “he [father] is more of the practical side, like ‘oh okay, then we'll do something about it now’, sometimes that is not necessary, but then you just want to get it off your chest.” Mothers were known to offer their help, such as Indira mentioned: “Yeah, then she says it's not bad at all and if you want to work on it, then we can work on it together and if not, then just leave it for a while until you feel chill and then by then we'll look at it', something like that.”

Adolescents appreciated that siblings, friends, partners, and professionals provided space for them to express their feelings following self‐disclosure. Friends, in particular, were recognized for validating adolescents' emotions and showing empathy. Adolescents also mentioned that professionals took specific actions to help them in response to self‐disclosure, such as learning how to challenge their thoughts or providing therapy. Although these actions were appreciated in most cases, some highlighted that an emotional or supportive response (in addition to the practical response) would have been appreciated. Indira, for instance, indicated that her teacher proposed to schedule a meeting with her classmates when she shared with the teacher that she was bullied: *“*…a little more compassion would have been nice. But on the other hand, she [the teacher] could not have done anything else about it.”

#### 3.2.4 Parents

##### 3.2.4.1 Parents' Self‐Disclosure

As fourth question, we aimed to gain insight into the perspectives of parents on their own self‐disclosure and the impact on their child's self‐disclosure. When it comes to parents' own self‐disclosure about distressing topics, mothers preferred to discuss their issues first with their friends, followed by their partners. Fathers often confided in their partners. Both mothers and fathers also coped by seeking distractions (such as reading books or using social media), exercising, talking with their own parent, and sharing with colleagues. Less frequently, they mentioned analyzing the problem, solving it independently, accepting it, and reading about it. Additionally, parents indicated that they sometimes withdrew, concealed their feelings, and moved on. Interestingly, some parents revealed that they were in the process of learning to communicate when they were not feeling well, acknowledging that this did not come naturally. The mother of Sofia stated: “Well I have some really good girlfriends I can talk with. Fortunately, yes. Depending on the topic, of course also with my husband. […] And I withdraw. Personally, I dive into books for example. At the moment I have a TikTok mania. […] By doing so, I try to suppress my unpleasant feelings.” And the father of Amy acknowledged that it may be a bad example: “I don't put it on the family. I withdraw. In that sense, I am a bad example.”

##### 3.2.4.2 Being Direct in Relation to Adolescents' Self‐Disclosure

Fathers indicated that “being direct” was a facilitating factor for their child to talk to them. For example, the father of Omar mentioned: “I ask fewer questions. Usually I'm more concise and straight to the point: like what's the problem.” However, Olivia's father also mentioned this as a barrier to talk: “Yes, our child would probably go to her mother rather than to me, I think. Because, yeah, I am a bit more direct than mother. That might be hard for my child.”

##### 3.2.4.3 Parents' Dilemma: Involvement or Privacy?

All parents indicated that they struggled with the dilemma of whether or not to approach their child when they sensed something was wrong, or whether to give them space to process and let them take the initiative to reach out, allowing them privacy. Parents noted that their child's angry or irritated responses, or a lack of visible emotions, made them hesitant about how to respond or approach. The father of Sofia stated: “I want to give her a hug, but on the other hand I also want to give her the space she needs.”

##### 3.2.4.4 Discrepancies Between Parents' and Adolescents' Reports of Support Figures

In addition to the research questions, we were able to the views of compare parents and adolescents at the family level, since we asked parents to indicate to whom they thought their adolescent would disclose. Both fathers and mothers thought that adolescents would seek support primarily from their mothers, with friends and fathers coming in a close second. Siblings and psychologists were also identified as important sources of support for adolescents. In all families, there was at least some overlap between parents' and adolescents' reports of whom adolescents talked to about these issues. However, in most families (8 of 11, 72.3%) for which we had information from both the adolescent and the parents, there were differences between the adolescents and their parents. For instance, adolescents' self‐disclosure to fathers was underestimated by both mothers and fathers. Moreover, both parents underestimated how much adolescents shared with their sibling(s). Adolescents' self‐disclosure to friends was over‐ and underestimated by parents. Some parents thought their adolescent shared a lot with friends, while this was not the case, and vice versa: Some parents thought that their adolescent did not share personal information with friends, while the adolescent indicated being very open with friends.

## 4 Discussion

The overarching aim of this study was to explore an essential skill for maintaining relationships and well‐being during adolescence: The ability to seek for social support through self‐disclosure (Tilton‐Weaver et al. 2014; Vijayakumar and Pfeifer 2020). Using a convergent parallel mixed methods design, we examined to whom adolescents disclose distressing topics, and investigated facilitators and barriers for doing so, or not. Quantitative analyses in 12 adolescents allowed us to gain some insight into whom adolescents disclose to. Based on these insights, we used qualitative information to reveal facilitators and barriers of adolescent self‐disclosure to caregivers, peers, relatives and familiar adults, professionals, and relevant others. The quantitative data show us the surface of existing patterns of adolescent self‐disclosure. Combining this data with a qualitative approach provides insight into the behavioral patterns that led to such frequencies. By also interviewing 23 parents, we were able to gain insight into parents' perspectives on their own self‐disclosure and its impact on adolescents' self‐disclosure. Quantitative results indicated that adolescents disclosed the most to their mothers, followed by friends, psychologists, and fathers. Qualitative analyses revealed two important facilitators were: (1) creating an opportunity to talk or share (i.e., a conversation was initiated or by spending time together); (2) the warm relationship with the conversation partner. Two important barriers were: (1) emotional distance; (2) privacy. Parents mentioned the dilemma of autonomy relatedness: approaching the adolescent versus giving space for the child to reach out for support.

### 4.1 Parents, Friends, and Psychologists, as Main Pillars for Adolescents' Self‐Disclosure on Distressing Topics

Adolescents in our study (ages 15–21) revealed that parents, especially mothers, are the person(s) they talk to the most about personal topics, such as school, health, family, and their body. This underscores the enduring role of parents as crucial sources of support (De Jonge et al. 2022), even when friends and peer relations become more important during adolescence (Flynn et al. 2017). At the same time, the important role of friends was also evident, as adolescents mentioned friends as important confidants with whom they talked about emotional and sensitive topics. Our findings on the frequency patterns of self‐disclosure contrasts with a previous quantitative study in a large sample of adolescents, which indicated that best friends were the most frequently disclosed confidants, followed by mothers and fathers (Elsharnouby and Dost‐Gözkan 2020). While some previous work has indicated has that some adolescents prefer to disclose to familiar adults rather than friends (Chen and Danish 2010), adolescents in our study hardly mentioned familiar adults as well as other persons who adolescents see every day (e.g., teachers, mentors), suggesting an under‐utilized opportunity for supporting adolescents. Similarly, internet and social media have increasingly become a place where adolescents spend a lot of time and may also connect with others to disclose their feelings and worries (Leijdesdorff et al. 2021; Luo and Hancock 2020). However, few adolescents in our study reached out to others online and only when they already had a connection.

### 4.2 Facilitators for Self‐Disclosure: A Created Opportunity and Warm Relationship

Several factors facilitated adolescents' self‐disclosure, and may help to explain why caregivers and peers were most frequently chosen as confidant. First, adolescents mentioned the importance of others initiating conversations and providing opportunities to talk or share (i.e., asking a question or by spending time together). Adolescents have more opportunities to disclose to caregivers and peers because they spend considerably more time with them than with a teacher or other familiar adults. This aligns with earlier findings that adolescents who spend more time with their parents are more likely to disclose more personal matters (Keijsers et al. 2010). In addition, adolescents tend to spend more time with mothers than with fathers (Janssen, Verkuil, van Houtum et al. 2024; Larson and Richards 1991), which may explain why adolescents report more opportunities for self‐disclosure to mothers. Adolescents in our study specifically mentioned spending less time with fathers as a reason for sharing less with them. Additionally, when caregivers or other familiar adults asked specific questions, adolescents were more likely to disclose, though this was not always the case. This suggests that fostering self‐disclosure through targeted questioning may be an effective approach, which is consistent with previous findings that both adolescents' active self‐disclosure and active inquiry of parents about emotions result in less emotional distress (Ding et al. 2022).

The second key facilitator of adolescents' self‐disclosure to caregivers and peers is the presence of a warm relationship—characterized by feelings of closeness—consistent with previous findings (Collins and Miller 1994; Villalobos Solís et al. 2015; Tilton‐Weaver et al. 2014). Self‐disclosure is not only supported by relational warmth but is also known as a core component in the formation and maintenance of interpersonal relationships (Willems et al. 2020). This suggest a reciprocal process: Warm relational bonds promote self‐disclosure, which in turn fosters intimacy and trust—elements that are crucial for developing and maintaining close relationships, particularly with peers and romantic partners (Finkenauer and Buyukcan‐Tetik 2015). In the parent–child relationship, self‐disclosure is suggested to play a less central role in relationship building than in more horizontal relationships (such as with peers or a partner; Tilton‐Weaver et al. 2014). Instead, the parent–child bond tends to be more strongly rooted in longstanding interaction patterns and established familial roles, rather than in (reciprocal) self‐disclosure. Nevertheless, across confidants, adolescents' feelings of warmth and closeness appear to facilitate their willingness to share personal experiences, underscoring the importance of emotional connectedness in self‐disclosure.

### 4.3 Barriers of Self‐Disclosure: Emotional Distance and Privacy Hindered Adolescent Self‐Disclosure

Adolescents identified several barriers that hindered their self‐disclosure. Emotional distance was one of them. This suits the reinforcing process between self‐disclosure and closeness. In the case of a less warm relationship, or more emotional distance, self‐disclosure may be(come) less, which in turn may also negatively impact the quality and closeness of the relationship (Willems et al. 2020). Emotional distance was particularly mentioned as a barrier in the context of self‐disclosure to members of their broader networks: teachers, relatives, and familiar adults. This contrasts with earlier work showing that some adolescents prefer neutral, non‐parental adults such as approachable psychologists at school (Chen and Danish 2010; Leijdesdorff et al. 2021). What may not help is that teachers may be hesitant to ask adolescents about emotional or sensitive topics. They may feel ill‐equipped to address such issues or believe these topics/conversations require professional expertise they lack (Timimi and Timimi 2022). However, avoiding these conversations or not instigating them may prevent the possibility of forming a trusted alliance in schools. Instead, adolescents primarily confided in teachers for practical, rather than emotional reasons, such as explaining absences (e.g., attending a funeral). Hence, building a warm and trusting relationship, starting with small gestures like asking questions or showing interest, could help to bridge the emotional gap.

In their most close relationships, an important barrier for adolescent self‐disclosure to caregivers and peers also lies within the adolescent. Certain topics felt too personal to share, and adolescents wanted to keep them private as these matters were not “their parents' or friends' business.” This aligns with the normative developmental process of adolescents learning to manage the sharing of personal information and the establishment of privacy boundaries (Camara et al. 2017; Petronio 2002; Smetana et al. 2006). In the context of the parent–adolescent relationship, adolescents and their parents may have different perceptions of what is private and what is routine information. Parents may believe that certain information is routine and that they should know about romantic partners of the adolescent, whereas the adolescent see this as private information (Marshall et al. 2005; Tilton‐Weaver et al. 2014). While much of the existing literature has examined adolescent privacy in relation to parents or family systems (Dietvorst et al. 2018; Petronio 2010), our findings suggest that privacy concerns also play a role in adolescents' willingness to disclose distressing topics to friends.

Shame emerged as another barrier to discussing distressing topics in almost all relationships. While privacy concerns can be seen as a normative aspect of adolescent development, shame may represent a more internalized and emotionally driven obstacle (Reimer 1996; Zeman et al. 2006). Previous work on barriers and facilitators to help‐seeking behavior for mental health problems, also indicated that shame was an important barrier to discussing mental health issues (Leijdesdorff et al. 2021; Radez et al. 2021). It only felt acceptable for adolescents to seek help when their problem was severe enough. Adolescents in our study echoed this, describing how they often felt that their problems were not serious enough to warrant discussion. This perception hindered them from talking about it with the people in their personal network.

### 4.4 How to Support Youth Best?

Adolescent self‐disclosure is inherently a reciprocal process with others, and these relationship dynamics may be essential to understand. Therefore, for our third question, we explored how adolescents perceive the responses from others to their self‐disclosure. In our study, adolescents emphasized the importance of socioaffective support that provides comfort and validation, such as the other person responding emphatically and being there for them. This finding is consistent with previous work (e.g., Barker 2007; Griffiths et al. 2011). Some literature suggests that incorporating a cognitive component, such as challenging thoughts by offering a different perspective or advice, may be essential for long‐term recovery following self‐disclosure (Nils and Rimé 2012). Instead, a very strong focus on socio‐affective support, without the needed cognitive component, may result in dwelling about negative feelings (i.e., co‐rumination) or relationship erosion, a process in which sharing of negative feelings may eventually lead to deterioration of social relationships (Coyne 1976; Stice et al. 2004). However, whereas all adolescents indicated that being seen and heard is essential for them to feel validated, they found cognitive support, such as advice or practical assistance, helpful only in specific situations, such as those related to schoolwork. In fact, a previous study has shown that receiving only cognitive support is strongly disliked by adolescents when expressing emotions such as anger and sadness (Pauw et al. 2018). Thus, while adolescents in our study expressed a stronger preference for socio‐affective support, the existing literature suggests that a balanced combination of both types of responses may ultimately foster adolescent well‐being and relationships in the long run.

Our findings tentatively suggest that adolescents turn to different persons, for either socioaffective or cognitive support. For instance, some adolescents in our study indicated that they would turn to their mothers for socio‐affective support (i.e., being listened to) and to their fathers for advice or a more practical advice or solutions. This aligns with the general notion that mothers may provide more emotional support and fathers give more instrumental care (Youniss and Smollar 1985), and that fathers are turned to for practical problems (de Jonge et al. (2022). Fathers in the study also noted this difference themselves, acknowledging their tendency to offer problem‐solving strategies rather than emotional validation. Hence, rather than suggesting that every confidant should combine both ingredients, adolescents suggest that different types of support may come from distinct individuals.

### 4.5 Parent's Perspective on Self‐Disclosure and Dilemma: To Approach or Give Space?

Our fourth question concerned parents' perspectives on their own self‐disclosure to potential confidants and its perceived impact on adolescents' self‐disclosure. Some parents reported sharing personal matters with their partner or friends, while others described using other strategies when coping with emotion or distressing situations. When reflecting on the how their own behavior might influence the adolescent's self‐disclosure, some reported they were learning to disclose more to their confidants to set a good example for their child.

While adolescents emphasized the importance of receiving appropriate support, parents reported struggling with how and when to approach their child. Parents expressed a recurring dilemma: balancing respect for their child's privacy with the need to offer support when sensing something was wrong. As adolescents mature, their growing need for autonomy often requires a renegotiation of boundaries around private matters (Smetana et al. 2006). Research on boundary disturbances (Petronio 2002) indicates that (unintended) privacy violation can reduce adolescents' willingness to disclose, increase secrecy, and negatively affect parent–child relationships (Dietvorst et al. 2018; Petronio 2002). These findings highlight the importance of future research aimed at supporting parents in navigating this dilemma.

Parents' timing and the setting of the conversation may play a crucial role in creating opportunities for adolescent self‐disclosure. Optimizing support of the adolescent requires tailoring responses to adolescents' individual needs—a principle referred to as the match hypothesis (Cohen and Wills 1985)—and calls for a context‐sensitive approach. For example, one father in our study described learning not to immediately question his child when sensing distress, as this often triggered resistance and conflicts. Instead, he learned to wait for the child to initiate the conversation or chooses a calm moment when the child is in their bedroom to ask how they are. Given the variability across individuals and situations, this process of matching support to the adolescent's needs is often one of trial and error. Based on our findings, we encourage parents to persist in their efforts to meet their child's needs, despite the challenges. Modeling self‐disclosure through sharing with trusted confidants may be one helpful strategy in fostering adolescents' willingness to disclose.

### 4.6 Strengths, Limitations, and Implications for Future Work

Given increasing pressure on adolescent mental health and the importance of self‐disclosure for obtaining support, this mixed methods study examined adolescents' self‐disclosure, along with its facilitators and barriers. A key strength of this study lies in that its broad focus on various potential confidants, with interviews offering in‐depth insights from both adolescent and parent perspective. However, the findings should be interpreted in the light of limitations. This study did not aim for generalization; we included a small and fairly homogeneous convenience sample of adolescents, all of whom had at least one person in whom they confided. Our focus was on who adolescents confided in, not on differences in disclosure across topics. While we offer some insights, future research could explore specific topic‐specific differences. Also, adolescent self‐disclosure may be effective at reducing depressive symptoms for adolescents (Gonsalves et al. 2023). However, in the current study with a small sample we were unable to assess the role of self‐disclosure in depression. Future research with larger samples and longitudinal designs are needed to examine whether self‐disclosure serves as a protective factor against depressive symptoms and to clarify the directionality of these associations.

The two most prominent facilitators for adolescents' self‐disclosure, namely the provision of concrete opportunities to talk and the warm relationship, have clear implications for interventions. Adolescents in our study suggested that spending time together and being asked questions can encourage self‐disclosure and strengthen relationships. These factors may be especially important for confidants who are currently often disregarded, such as teachers and relatives, because of the emotional distance that is experienced. Our findings highlight the importance of providing concrete opportunities, such as initiating conversations, that may reduce the emotional distance and facilitates adolescents to disclose. Creating such opportunities may also tackle the barrier of emotional distance experienced by adolescents. These suggestions align with the Capability, Opportunity, Motivation, Behavior (COM‐B) model, a leading behavioral science framework on behavior change (Michie et al. 2014). In accordance with the second and third pillars of the COM‐B model, our findings suggest several *opportunities* (e.g., spending time together) and *motivations* (e.g., the adolescents' emotional or practical need) that may foster self‐disclosure. However, this study did not address the *capabilities* of adolescents and their (potential) confidants (e.g., “can parents or teachers ask the right questions or meet the needs of adolescents”) in the context of self‐disclosure. Future work is needed to better understand what may be required to enhance adolescents' and confidants' capabilities to foster adolescent self‐disclosure.

To conclude, this mixed methods study showed that while adolescents frequently disclose to parents, friends, and psychologists, they share less with other familiar adults—an area warranting further attention. Self‐disclosure was facilitated by opportunities to talk and warm relationships, while emotional distance and uncertainty about when or what to share—especially with sensitive or shame‐related topics—emerged as barriers. Parents reported struggling to balance giving space with offering support. With regard to parents own self‐disclosure to confidants, modeling self‐disclosure (e.g., sharing personal experiences with a partner or friend) might be an important strategy for parents. Adolescents often seek comfort but prefer different confidants depending on the type of support needed (e.g., emotional vs*.* practical). Under the right conditions, they appear willing to share distressing topics, but familiar adults may need to be more active in creating opportunities for disclosure by making time, offering space, and initiating conversations.

## Author Contributions

Marie‐Louise J. Kullberg, Loes Keijsers, Bernet Elzinga, and Loes H. C. Janssen designed the study. Marie‐Louise J. Kullberg and Loes H. C. Janssen collected the data. Marie‐Louise J. Kullberg and Loes H. C. Janssen transcribed and analyzed the data. Marie‐Louise J. Kullberg, Loes Keijsers, Bernet Elzinga, and Loes H. C. Janssen wrote the manuscript. All authors contributed to the interpretation of results, revised the manuscript, and approved the final version.

## Funding

The study was supported by a personal research grant from de Nederlandse Organisatie voor Wetenschappelijk Onderzoek (NWO) awarded to Bernet Elzinga (VICI; Unravelling the Impact of Emotional Maltreatment on the Developing Brain 453‐15‐006) and by the European Research Council (ERC, PARADOx, 101043536). Views and opinions expressed are however those of the author(s) only and do not necessarily reflect those of the European Union or the European Research Council Executive Agency. Neither the European Union nor the granting authority can be held responsible for them.

## Ethics Statement

Ethical approval was obtained from Research Ethics Committee at Leiden University on October 15, 2021 (reference number 2021‐10‐12‐B.M. Elzinga‐V2‐3476).

## Consent

Consent from all participants was obtained. If adolescents were below 16, parents also provided informed consent for their child.

## Conflicts of Interest

The authors declare no conflicts of interest.

## Supporting information


**Appendix 1:** Matrix used to discuss with adolescents including topics and persons.
**Appendix 2:** Overview of how often (%) adolescents always self‐disclose to persons per topic and per person.
**Appendix 3:** Overview of how often (%) adolescents never self‐disclose to persons per topic and per person.
**Appendix 4:** Overview of how often (%) adolescents sometimes self‐disclose to persons per topic and per person.

## Data Availability

The authors have nothing to report.
