# Supplementary material for: Facilitators and Barriers of Adolescent Self‐Disclosure Across Different Confidants: A Multi‐Informant Mixed Methods Study
Source: J Adolesc. 2026 Jan 5;98(3):879–94. doi: 10.1002/jad.70100 (PMC13044866; doi:10.1002/jad.70100)
Supplement: Supplementary file 1 — Appendix 1: Matrix used to discuss with adolescents including topics and persons. Appendix 2: Overview of how often (%) adolescents always self‐disclose to persons per topic and per person. Appendix 3: Overview of how often (%) adolescents never self‐disclose to persons per topic and per person. Appendix 4: Overview of how often (%) adolescents sometimes self‐disclose to persons per topic and per person. [file JAD-98-879-s001.docx]

**Facilitators and barriers of adolescent self-disclosure: A multi-informant mixed method study**

Supplement

Appendix 1. Matrix used to discuss with adolescents including topics and persons

|  | Mother | Father | Sibling | Stepmother | Stepfather | Stepsibling | Uncle / aunt | Grandmother/ grandfather | Neighbors / family friends | Friend | Teacher / mentor | GP | Psychologist | Other professional | Others online | Searching information online | Other, namely |
| --- | --- | --- | --- | --- | --- | --- | --- | --- | --- | --- | --- | --- | --- | --- | --- | --- | --- |
| Health issues of a family member (father, mother, sibling, other family members) |  |  |  |  |  |  |  |  |  |  |  |  |  |  |  |  |  |
| Family issues (father, mother, sibling, other family members) |  |  |  |  |  |  |  |  |  |  |  |  |  |  |  |  |  |
| Financial problems |  |  |  |  |  |  |  |  |  |  |  |  |  |  |  |  |  |
| Disagreement regarding beliefs |  |  |  |  |  |  |  |  |  |  |  |  |  |  |  |  |  |
| Negative emotions |  |  |  |  |  |  |  |  |  |  |  |  |  |  |  |  |  |
| Mental health problems |  |  |  |  |  |  |  |  |  |  |  |  |  |  |  |  |  |
| Issues/Problems related to school |  |  |  |  |  |  |  |  |  |  |  |  |  |  |  |  |  |
| Bullying, exclusion, racism, intimidation |  |  |  |  |  |  |  |  |  |  |  |  |  |  |  |  |  |
| Issues with friends/friendships |  |  |  |  |  |  |  |  |  |  |  |  |  |  |  |  |  |
| Issues with partner/romantic relationships |  |  |  |  |  |  |  |  |  |  |  |  |  |  |  |  |  |
| Bodily issues |  |  |  |  |  |  |  |  |  |  |  |  |  |  |  |  |  |
| Identity issues |  |  |  |  |  |  |  |  |  |  |  |  |  |  |  |  |  |
| Social media issues |  |  |  |  |  |  |  |  |  |  |  |  |  |  |  |  |  |
| Health issues |  |  |  |  |  |  |  |  |  |  |  |  |  |  |  |  |  |
| Issues with alcohol, drugs, smoking, gaming |  |  |  |  |  |  |  |  |  |  |  |  |  |  |  |  |  |
| Other negative events (death of someone) |  |  |  |  |  |  |  |  |  |  |  |  |  |  |  |  |  |

Appendix 2. Overview of how often (%) adolescents always self-disclose to persons per topic and per person

|  | Health issues family | Family issues | Financial problems | Disagreement regarding beliefs | Negative emotions | Mental health problems | Issues related to school | Bullying, exclusion, racism, intimidation | Issues with friends | Issues with partner/ romantic relationship | Bodily issues | Identity issues | Social media issues | Health issues | Issues with alcohol, drugs, smoking, gaming | Other negative events |
| --- | --- | --- | --- | --- | --- | --- | --- | --- | --- | --- | --- | --- | --- | --- | --- | --- |
| Mother | 90.0 | 100.0 | 100.0 | 100.0 | 66.7 | 71.4 | 100.0 | 57.1 | 75.0 | 50.0 | 75.0 | 50.0 | 50.0 | 100.0 | 25.0 | 100.0 |
| Father | 66.7 | 50.0 | 75.0 | 85.7 | 50.0 | 14.3 | 80.0 | 42.9 | 57.1 | 0.0 | 28.6 | 0.0 | 20.0 | 40.0 | 25.0 | 80.0 |
| Sibling | 55.6 | 50.0 | 20.0 | 71.4 | 70.0 | 25.0 | 40.0 | 37.5 | 28.6 | 20.0 | 0.0 | 0.0 | 20.0 | 16.7 | 25.0 | 66.7 |
| Stepmother | 0.0 | 0.0 | 0.0 | 0.0 | 0.0 | 0.0 | 0.0 | 0.0 | 0.0 | 0.0 | 0.0 |  | 0.0 | 0.0 |  | 0.0 |
| Stepfather | 100.0 | 100.0 | 50.0 | 100.0 | 50.0 | 100.0 | 50.0 | 50.0 | 100.0 | 50.0 | 0.0 |  |  | 100.0 |  | 100.0 |
| Stepsibling | 0.0 | 0.0 | 0.0 | 0.0 | 0.0 | 0.0 | 0.0 | 0.0 |  | 0.0 | 0.0 |  | 0.0 | 0.0 |  | 0.0 |
| Aunt/uncle | 16.7 | 0.0 | 0.0 | 0.0 | 0.0 | 0.0 | 0.0 | 0.0 | 0.0 | 0.0 | 0.0 | 0.0 | 25.0 | 0.0 | 0.0 | 25.0 |
| Grandmother/ grandfather | 44.4 | 0.0 | 0.0 | 12.5 | 10.0 | 0.0 | 0.0 | 0.0 | 0.0 | 0.0 | 0.0 | 0.0 | 0.0 | 0.0 | 0.0 | 50.0 |
| Neighbors/ family acquaintances | 0.0 | 0.0 |  | 0.0 | 0.0 | 0.0 | 0.0 | 0.0 | 0.0 | 0.0 | 0.0 |  | 0.0 | 0.0 | 0.0 |  |
| Friends | 60.0 | 22.2 | 0.0 | 37.5 | 36.4 | 50.0 | 54.5 | 50.0 | 62.5 | 100.0 | 22.2 | 50.0 | 100.0 | 50.0 | 100.0 | 33.3 |
| Teacher/ mentor | 30.0 | 11.1 | 0.0 | 0.0 | 18.2 | 11.1 | 50.0 | 22.2 | 0.0 | 0.0 | 0.0 | 0.0 | 0.0 | 0.0 | 0.0 | 16.7 |
| GP | 0.0 | 0.0 | 0.0 | 0.0 | 10.0 | 75.0 | 0.0 | 0.0 | 0.0 | 0.0 | 12.5 | 0.0 | 0.0 | 50.0 | 0.0 | 16.7 |
| Psychologist | 57.1 | 62.5 | 33.3 | 33.3 | 87.5 | 100.0 | 87.5 | 66.7 | 14.3 | 60.0 | 14.3 | 0.0 | 0.0 | 66.7 | 33.3 | 60.0 |
| Other professionals | 50.0 | 50.0 | 100.0 | 0.0 | 33.3 | 50.0 | 50.0 | 50.0 | 0.0 | 50.0 | 25.0 |  | 0.0 | 50.0 | 0.0 | 50.0 |
| Other online | 0.0 | 0.0 | 0.0 | 0.0 | 0.0 | 0.0 | 0.0 | 0.0 | 11.1 | 0.0 | 0.0 | 0.0 | 0.0 | 0.0 | 0.0 | 0.0 |
| Search information online | 33.3 | 0.0 | 0.0 | 37.5 | 10.0 | 50.0 | 0.0 | 25.0 | 0.0 | 16.7 | 44.4 | 50.0 | 20.0 | 66.7 | 0.0 | 16.7 |
| Other* | 57.1 | 50.0 | 60.0 | 57.1 | 66.7 | 33.3 | 75.0 | 40.0 | 85.7 | 66.7 | 0.0 | 50.0 | 25.0 | 60.0 | 100.0 | 50.0 |

*Note.* The other category included for instance, partner, colleague, classmate, the person I am dating, God. Percentages are controlled for the not applicable option, thus adolescents were left out of the calculation when they did not worry about a topic or when they did not have contact with a certain person. Empty cells indicate that person and topic were not applicable for all adolescents.

Appendix 3. Overview of how often (%) adolescents never self-disclose to persons per topic and per person

|  | Health issues family | Family issues | Financial problems | Disagreement regarding beliefs | Negative emotions | Mental health problems | Issues related to school | Bullying, exclusion, racism, intimidation | Issues with friends | Issues with partner/ romantic relationship | Bodily issues | Identity issues | Social media issues | Health issues | Issues with alcohol, drugs, smoking, gaming | Other negative events |
| --- | --- | --- | --- | --- | --- | --- | --- | --- | --- | --- | --- | --- | --- | --- | --- | --- |
| Mother | 10.0 | 0.0 | 0.0 | 0.0 | 27.3 | 12.5 | 0.0 | 37.5 | 12.5 | 16.7 | 25.0 | 50.0 | 20.0 | 0.0 | 50.0 | 0.0 |
| Father | 0.0 | 0.0 | 0.0 | 0.0 | 30.0 | 14.3 | 10.0 | 42.9 | 28.6 | 60.0 | 57.1 | 50.0 | 40.0 | 0.0 | 75.0 | 0.0 |
| Sibling | 0.0 | 37.5 | 60.0 | 0.0 | 20.0 | 50.0 | 30.0 | 50.0 | 28.6 | 60.0 | 71.4 | 50.0 | 40.0 | 33.3 | 25.0 | 16.7 |
| Stepmother | 50.0 | 0.0 | 100.0 | 100.0 | 100.0 | 0.0 | 50.0 | 100.0 | 0.0 | 50.0 | 100.0 |  | 100.0 | 100.0 |  | 50.0 |
| Stepfather | 0.0 | 0.0 | 0.0 | 0.0 | 0.0 | 0.0 | 50.0 | 50.0 | 0.0 | 50.0 | 50.0 |  |  | 0.0 |  | 0.0 |
| Stepsibling | 50.0 | 100.0 | 100.0 | 100.0 | 100.0 | 100.0 | 100.0 | 100.0 |  | 100.0 | 100.0 |  | 100.0 | 100.0 |  | 100.0 |
| Aunt/uncle | 33.3 | 80.0 | 100.0 | 100.0 | 100.0 | 80.0 | 66.7 | 100.0 | 100.0 | 75.0 | 100.0 | 100.0 | 50.0 | 66.7 | 33.3 | 50.0 |
| Grandmother/ grandfather | 33.3 | 50.0 | 100.0 | 62.5 | 80.0 | 57.1 | 60.0 | 71.4 | 85.7 | 83.3 | 100.0 | 100.0 | 75.0 | 80.0 | 100.0 | 50.0 |
| Neighbors/ family acquaintances | 100.0 | 100.0 |  | 100.0 | 100.0 | 100.0 | 100.0 | 100.0 | 100.0 | 100.0 | 100.0 |  | 100.0 | 100.0 | 100.0 |  |
| Friends | 20.0 | 33.3 | 20.0 | 50.0 | 18.2 | 25.0 | 9.1 | 25.0 | 25.0 | 0.0 | 44.4 | 50.0 | 0.0 | 33.3 | 0.0 | 16.7 |
| Teacher/ mentor | 40.0 | 77.8 | 83.3 | 85.7 | 72.7 | 55.6 | 30.0 | 22.2 | 100.0 | 100.0 | 100.0 | 66.7 | 80.0 | 66.7 | 100.0 | 50.0 |
| GP | 100.0 | 100.0 | 100.0 | 85.7 | 80.0 | 12.5 | 90.0 | 100.0 | 85.7 | 83.3 | 87.5 | 100.0 | 100.0 | 50.0 | 75.0 | 66.7 |
| Psychologist | 14.3 | 25.0 | 33.3 | 50.0 | 0.0 | 0.0 | 0.0 | 33.3 | 57.1 | 0.0 | 42.9 | 0.0 | 80.0 | 16.7 | 33.3 | 40.0 |
| Other professionals | 50.0 | 50.0 | 0.0 | 66.7 | 33.3 | 50.0 | 50.0 | 50.0 | 50.0 | 0.0 | 75.0 |  | 100.0 | 50.0 | 0.0 | 50.0 |
| Other online | 81.8 | 100.0 | 100.0 | 77.8 | 81.8 | 88.9 | 100.0 | 100.0 | 88.9 | 100.0 | 100.0 | 100.0 | 100.0 | 100.0 | 100.0 | 100.0 |
| Search information online | 22.2 | 77.8 | 80.0 | 50.0 | 50.0 | 12.5 | 80.0 | 75.0 | 85.7 | 83.3 | 22.2 | 0.0 | 40.0 | 16.7 | 50.0 | 83.3 |
| Other* | 0.0 | 50.0 | 20.0 | 28.6 | 11.1 | 50.0 | 25.0 | 60.0 | 14.3 | 16.7 | 71.4 | 50.0 | 50.0 | 20.0 | 0.0 | 33.3 |

*Note.* The other category included for instance, partner, colleague, classmate, the person I am dating, God. Percentages are controlled for the not applicable option, thus adolescents were left out of the calculation when they did not worry about a topic or when they did not have contact with a certain person. Empty cells indicate that person and topic were not applicable for all adolescents.

Appendix 4. Overview of how often (%) adolescents sometimes self-disclose to persons per topic and per person

|  | Health issues family | Family issues | Financial problems | Disagreement regarding beliefs | Negative emotions | Mental health problems | Issues related to school | Bullying, exclusion, racism, intimidation | Issues with friends | Issues with partner/ romantic relationship | Bodily issues | Identity issues | Social media issues | Health issues | Issues with alcohol, drugs, smoking, gaming | Other negative events |
| --- | --- | --- | --- | --- | --- | --- | --- | --- | --- | --- | --- | --- | --- | --- | --- | --- |
| Mother | 0.0 | 33.3 | 0.0 | 12.5 | 18.2 | 25.0 | 18.2 | 12.5 | 12.5 | 50.0 | 0.0 | 0.0 | 40.0 | 0.0 | 25.0 | 0.0 |
| Father | 33.3 | 50.0 | 25.0 | 14.3 | 20.0 | 71.4 | 10.0 | 14.3 | 14.3 | 40.0 | 14.3 | 50.0 | 40.0 | 60.0 | 0.0 | 20.0 |
| Sibling | 44.4 | 12.5 | 20.0 | 28.6 | 10.0 | 25.0 | 30.0 | 12.5 | 42.9 | 20.0 | 28.6 | 50.0 | 40.0 | 50.0 | 50.0 | 16.7 |
| Stepmother | 50.0 | 100.0 | 0.0 | 0.0 | 0.0 | 100.0 | 50.0 | 0.0 | 100.0 | 50.0 | 0.0 |  | 0.0 | 0.0 |  | 50.0 |
| Stepfather | 0 | 0 | 50 | 0 | 50 | 0 | 0 | 0 | 0 | 0 | 50 |  |  | 0 |  | 0 |
| Stepsibling | 50 | 0 | 0 | 0 | 0 | 0 | 0 | 0 |  | 0 | 0 |  | 0 | 0 |  | 0 |
| Aunt/uncle | 50.0 | 20.0 | 0.0 | 0.0 | 0.0 | 20.0 | 33.3 | 0.0 | 0.0 | 25.0 | 0.0 | 0.0 | 25.0 | 33.3 | 66.7 | 25.0 |
| Grandmother/ grandfather | 22.2 | 50.0 | 0.0 | 25.0 | 10.0 | 42.9 | 40.0 | 28.6 | 14.3 | 16.7 | 0.0 | 0.0 | 25.0 | 20.0 | 0.0 | 0.0 |
| Neighbors/ family acquaintances | 0 | 0 |  | 0 | 0 | 0 | 0 | 0 | 0 | 0 | 0 |  | 0 | 0 | 0 |  |
| Friends | 20.0 | 44.4 | 80.0 | 12.5 | 45.5 | 25.0 | 36.4 | 25.0 | 12.5 | 0.0 | 33.3 | 0.0 | 0.0 | 16.7 | 0.0 | 50.0 |
| Teacher/ mentor | 30.0 | 11.1 | 16.7 | 14.3 | 9.1 | 33.3 | 20.0 | 55.6 | 0.0 | 0.0 | 0.0 | 33.3 | 20.0 | 33.3 | 0.0 | 33.3 |
| GP | 0.0 | 0.0 | 0.0 | 14.3 | 10.0 | 12.5 | 10.0 | 0.0 | 14.3 | 16.7 | 0.0 | 0.0 | 0.0 | 0.0 | 25.0 | 16.7 |
| Psychologist | 28.6 | 12.5 | 33.3 | 16.7 | 12.5 | 0.0 | 12.5 | 0.0 | 28.6 | 40.0 | 42.9 | 100.0 | 20.0 | 16.7 | 33.3 | 0.0 |
| Other professionals | 0.0 | 0.0 | 0.0 | 33.3 | 33.3 | 0.0 | 0.0 | 0.0 | 50.0 | 50.0 | 0.0 |  | 0.0 | 0.0 | 100.0 | 0.0 |
| Other online | 18.2 | 0.0 | 0.0 | 22.2 | 18.2 | 11.1 | 0.0 | 0.0 | 0.0 | 0.0 | 0.0 | 0.0 | 0.0 | 0.0 | 0.0 | 0.0 |
| Search information online | 44.4 | 22.2 | 20.0 | 12.5 | 40.0 | 37.5 | 20.0 | 0.0 | 14.3 | 0.0 | 33.3 | 50.0 | 40.0 | 16.7 | 50.0 | 0.0 |
| Other* | 42.9 | 0.0 | 20.0 | 14.3 | 22.2 | 16.7 | 0.0 | 0.0 | 0.0 | 16.7 | 28.6 | 0.0 | 25.0 | 20.0 | 0.0 | 16.7 |

*Note.* The other category included for instance. partner. colleague. classmate. the person I am dating. God. Percentages are controlled for the not applicable option. thus adolescents were left out of the calculation when they did not worry about a topic or when they did not have contact with a certain person. Empty cells indicate that person and topic were not applicable for all adolescents.
